# The ant genus *Carebara* Westwood in the Arabian Peninsula (Hymenoptera, Formicidae)

**DOI:** 10.3897/zookeys.357.5946

**Published:** 2013-12-02

**Authors:** Mostafa R. Sharaf, Abdulrahman S. Aldawood

**Affiliations:** 1 Plant Protection Department, College of Food and Agriculture Sciences, King Saud University, Riyadh 11451, PO Box 2460, Kingdom of Saudi Arabia

**Keywords:** Saudi Arabia, Palearctic region, Myrmicinae, key, taxonomy, new species

## Abstract

The ant genus *Carebara* of the Arabian Peninsula is revised. *Carebara abuhurayri* Sharaf & Aldawood, 2011 is synonymized under *Carebara arabica* Collingwood & van Harten, 2001. *Carebara arabica* is redescribed and a Neotype is fixed based on a specimen collected from southwestern Kingdom of Saudi Arabia. A new species, *C. fayrouzae* **sp. n.** is described from Saudi Arabia based on queens, major and minor workers. Keys to major and minor workers of the two Arabian *Carebara* species are given.

## Introduction

The ant genus *Carebara* Westwood, 1840, *sensu* Fernández (2004), contains more than 180 described species (Bolton et al. 2006) and is distributed worldwide in the subtropics and tropics with regional taxonomic treatments available for the Palearctic (Ettershank 1966, Weber 1950, Xu 1999, Guénard and Dunn 2012), Afrotropical including Madagascar (Weber 1950, Brown 2000, Taylor 2010, Frank Azorsa, unpublished data), Neotropics (Brown 2000), Indo-Malayan (Bingham 1903), Oriental (Terayama 1996, Terayama 2009) and Australian (Taylor and Brown 1985, Shattuck 1999). Hitherto, Fernández (2004) remains the most important and comprehensive treatment of the genus for the Western Hemisphere. Five genera, *Oligomyrmex* Mayr,1867, *Paedalgus* Forel, 1911, *Afroxyidris* Belshaw & Bolton, 1994, *Parvimyrma*, Eguchi & Bui, 2007 and *Neoblepharidatta* Sheela & Narendran, 1997 have been synonymized under *Carebara* (Fernández 2004).

However, very little taxonomic or biological information is available on the genus *Carebara* throughout its range (Bharti and Kumar 2013), especially in the Arabian Peninsula (Aldawood et al. 2011). The scarcity of information may be due to the cryptic nature of species, tiny body size, and the difficulty in collecting these ants requiring leaf litter sifting and the use of Berlese funnels for extraction. Members of the genus are subterranean and often associated with decaying wood and leaf litter (Bolton 1973, Longino 2004, Aldawood et al. 2011, Bharti and Kumar 2013).

*Carebara* was originally recorded from the Arabian Peninsula by Collingwood and van Harten (2001) with their description of *Oligomyrmex arabicus* based on minor and major workers collected from Al Kawd, near Abyan, Republic of Yemen. Ten years later, we described a new species of *Carebara*, *Carebara abuhurayri* Sharaf & Aldawood based on minor workers from the southwestern mountains of Kingdom of Saudi Arabia (KSA) (Aldawood et al. 2011).

Several nest series of a species very similar to *Carebara arabica* were collected from four different localities in the southwestern region of KSA. Minor and major workers matched the brief original description of *Carebara arabica*. In addition, two major workers of *Carebara abuhurayri* were collected from its type locality and are very similar to the major workers of *Carebara arabica*. Further comparisons of this newly collected material indicated that *Carebara abuhurayri* is a synonym of *Carebara arabicus*.

Minor workers of another *Carebara* species that appeared to be undescribed were collected from Riyadh, KSA. Repeated efforts to find nests of this species that contained all castes were unsuccessful; however, a colony that contained minor and major workers and several alate queens (males unknown) was collected in eastern KSA, confirming the novelty of this taxon.

In the present work, a new species, *Carebara fayrouzae* sp. n., is described based on queens, major, and minor workers. *Carebara arabica* is redescribed anddetailed new measurements are given. A Neotype of *Carebara arabica* from a locality in KSA Arabia near the Republic of Yemen is designated. *Carebara abuhurayri* is synonymized with *Carebara arabica*. Keys to major and minor workers of the two known Arabian Peninsula species are given.

## Material and methods

### Measurements and indices

TL Total Length (HL+ Mandible length+ ML + Petiole Length + Postpetiole length + Gaster length)

HW Head Width; maximum width of head behind eyes in full face view

HL Head Length; maximum length of head, excluding mandibles

SL Scape Length; excluding basal neck

EL Eye Length; maximum diameter of eye

ML Mesosoma Length; length of mesosoma in lateral view, from the point at which pronotum meets cervical shield to posterior base of propodeal lobes or teeth

PRW Pronotal width, maximum width in dorsal view

PL Petiole Length; maximum length measured in dorsal view, from anterior margin to posterior margin

PW Petiole Width; maximum width measured in dorsal view

PPL Postpetiole Length; maximum length measured in dorsal view

PPW Postpetiole Width; maximum width measured in dorsal view

#### Indices:

SI Scape Index (SL × 100/HW)

CI Cephalic Index (HW × 100/HL)

All measurements are in millimeters and follow the standard measurements of Fernández (2004).

#### Acronyms of museums:

BMNH Natural History Museum, London, United Kingdom

CASC California Academy of Science Collection, San Francisco, California, USA

KSMA King Saud University Museum of Arthropods, King Saud University, Riyadh, KSA

MCZC Museum of Comparative Zoology, Harvard University, Cambridge, MA, USA

MHNG Muséum ďHistoire Naturelle, Geneva, Switzerland

NHMB Naturhistorisches Museum, Basel, Switzerland

SEMC Division of Entomology (Snow Entomological Collections), University of Kansas Natural History Museum, Lawrence, Kansas, USA

WMLC World Museum Liverpool, Liverpool, United Kingdom

## Results

### Key to Arabian *Carebara*

#### Major worker

**Table:** 

1	Smallerspecies (TL 1.77–2.76); antennae 10-segmented; concolorous brownish, antennae and legs yellowish; posterior margin of head strongly concave and posterior corners with a pair of teeth or horns, appearing blunt in profile; cephalic dorsum dull, with fine, dense, regular and longitudinal rugulae; lateral margins of postpetiole in dorsal view rounded	*Carebara arabica*
–	Largerspecies (TL 3.27–5.00); antennae 9-segmented; bicolored, head and mesosoma brownish, petiole and postpetiole brownish yellow, antennae, legs and gaster clear yellowish; posterior margin of head feebly concave and posterior corners rounded, without teeth or horns; cephalic dorsum smooth and shining except anterior part of head finely, longitudinally rugulose; lateral margins of postpetiole distinctly angular in dorsal view	*Carebara fayrouzae* sp. n.

#### Minor worker

**Table:** 

1	Antennae 10-segmented; eyes minute, with a single ommatidium (present in all individuals); body pilosity subdecumbent or appressed and much scarce; anterolateral sides of head very finely longitudinally striated; lower halves of mesopleuron, metapleuron, petiole and postpetiole areolate-rugose; propodeal dorsum nearly half as long as propodeal declivity in profile	*Carebara arabica*
–	Antennae 9-segmented; eyes as rudimentary ommatidium (absent in some individuals); body pilosity erect to suberect and dense; entire body smooth without any type of surface sculpture; propodeal dorsum as long as declivity, appearing as a continuous curve in profile	*Carebara fayrouzae* sp. n.

### Carebara arabica

(Collingwood & van Harten, 2001)

http://species-id.net/wiki/Carebara_arabica

Figs 1 –6

Oligomyrmex arabica Collingwood & van Harten, 2001: 564, figs 2–4 (s. w.). **Neotype** major worker. SAUDI ARABIA, Almajardah, Wadi Khat, 10.xi.2012, 19.08913°N, 41.97126°E, 513 m, by leaf litter sifting (M. R. Sharaf leg) (KSMA) (CASENT0906367). Holotype major worker, YEMEN, Al Kawd (misspelled AI Kowd), 13.088622°, 45.364722°, viii.1999, in light-trap, (van Harten & Al Haruri), paratypes, 7 minor workers, same data as holotype [not in WMLC, all presumably lost]. Combination in *Carebara*: new combination (unpublished) (Bolton 2012).
Carebara abuhurayri Sharaf & Aldawood, in Aldawood et al. 2011: 63, figs 1–12 (w). Holotype minor worker, SAUDI ARABIA, Al Bahah, Al Mukhwah, Zei Ein Archaeological Village (sometimes written Dhi Ain archaeological village), 19.91667°N, 41.43333°E, 741 m., 18.v.2010 (M. R. Sharaf Leg.), paratypes, 7 minor workers, same data as the holotype (KSMA) [examined]. Syn. n.

#### Additional material.

(3 major workers, 5 minor workers (CASENT0906368)) same data as the neotype; 6 major workers, 6 minor workers, SAUDI ARABIA, Wadi Bagara, 10.xi.2012, 18.79287°N, 42.01857°E, 436m, by leaf litter sifting (M. R. Sharaf leg.); 1 major worker, 9 minor workers, SAUDI ARABIA, Wadi Aljora, near Abadan, 12.xi.2012, 17.29263°N, 43.07010°E, 465 m, by leaf litter sifting (M. R. Sharaf leg.); 12 minor workers, SAUDI ARABIA, Fayfa, Agriculture Research Station, 6.iv.2013, 17.28671°N, 43.14390°E, 879m, (M. R. Sharaf leg.); 5 minor workers,SAUDI ARABIA, Fayfa, Agriculture Research Station, 5.iv.2013, 17.28671°N, 43.14390°E, 879m, (M. R. Sharaf leg.) **[KSMA]**; 1 major worker, SAUDI ARABIA, Al Bahah, Al Mukhwah, Zei Ein Archaeological Village, 19.9294°N, 41.4417°E, 741 m., 15.v.2011, (M. R. Sharaf Leg.); 1 major worker, SAUDI ARABIA, Al Bahah, Al Mukhwah, Dhi Ain Archaeological Village, 19.928°N, 41.4419°E ±50 m, 735 m., (B. L. Fisher Leg.), 23.ix.2011, Coll. Code BLF27577 **[CASC]**.

#### Description.

**Neotype major worker.** TL 2.45, HL 0.71, HW 0.52, SL 0.26, ML 0.59, PRW 0.35, PL 0.15, PW 0.17, PPL 0.12, PPW 0.21, SI 50, CI 73.

**Major workers.** TL 1.77-2.76, HL 0.56-0.72, HW 0.44-0.52, SL 0.22-0.28, ML 0.49-0.63, PRW 0.29-0.35, PL 0.12-0.19, PW 0.12-0.17, PPL 0.11-0.18, PPW 0.14-0.25, SI 48-64, CI 69-80 (N=10).

Holotype major worker: TL 2.53, HL 0.75, HW 0.36, SL 0.63 (Collingwood and van Harten 2001) [Presumably lost]. (In the original description, the HW and SL for major are given wrongly as 0.36 and 0.63 respectively, from the illustration they would be ca. HW 0.55 and SL 0.30).

**Figures 1–3.**
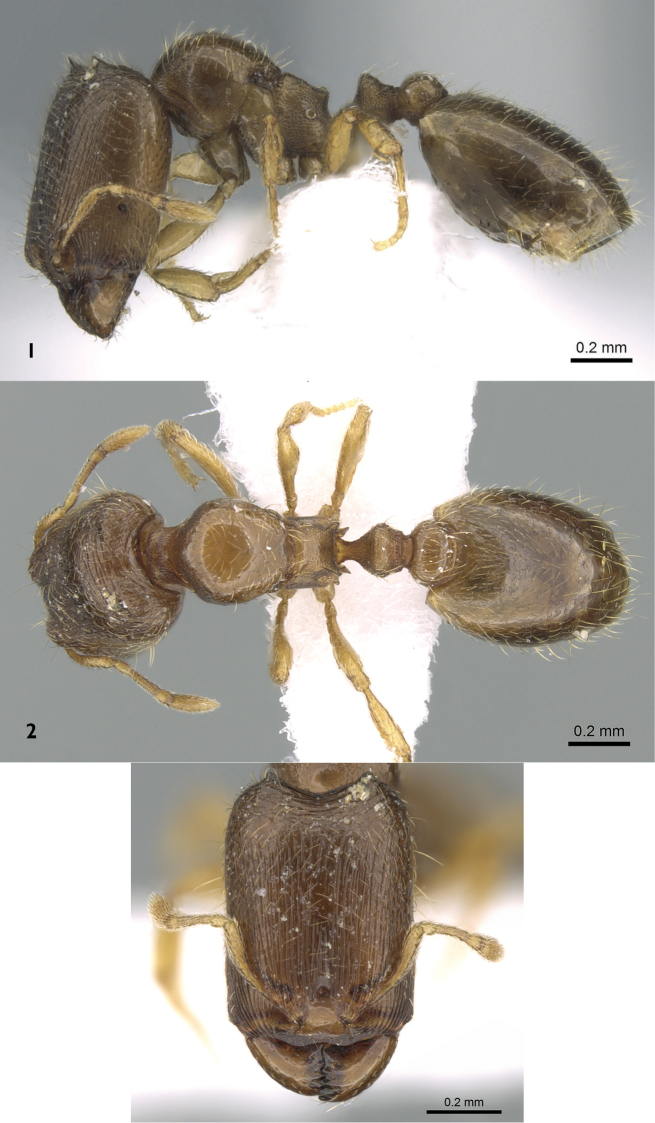
*Carebara arabica*, major worker. **1** body in profile **2** body in dorsal view **3** head in full-face view (antweb.org, CASENT0906367).

**Major worker.** (Figs 1–3) Headrectangular (HL ~ 1.38 × HW) with strongly concave posterior margin and straight parallel sides; mandibles smooth and shining; masticatory margin armed with five teeth; eyes with a single oval ommatidium; anterior clypeal margin shallowly concave; antennae ten segmented with a two segmented club; scapes very short (mean SI = 54); posterior margin of head transversally carinate and posterior corners with a pair of outgrowths, appearing as blunt teeth in lateral view. Promesonotum strongly convex; metanotal area with apparent vestigial wing bases; metanotal groove deep; propodeal spines blunt, short and broadly based; petiole distinctly broader than long in dorsal view. Postpetiole clearly broader than long and broader than petiole in dorsal view. Gaster smooth and shining. Sculpture: cephalic dorsum and area in front of eyes finely densely regularly longitudinally rugulose; the ground-sculpture a fine, dense, conspicuous granulation; lateral cephalic dorsum from the posterior margin of eyes to posterior margin of head faintly and densely granulate; promesonotum smooth and shining; anepisternum smooth and shining; katepisternum and propodeum densely, transversely and conspicuously reticulate-punctate; petiole densely irregularly reticulate; postpetiole dorsum smooth and shining. Pilosity: head hairs long and sparse; petiole with two pairs of long backward directed hairs; postpetiole with three pairs of long hairs; gaster with few scattered long suberect hairs and abundant subdecumbent short hairs. Colour:concolorous brownish.

**Minor workers.** TL 0.99–1.13, HL 0.35–0.41, HW 0.29–0.32, SL 0.21–0.28, ML 0.31–0.34, PRW 0.17–0.19, PL 0.08–0.12, PW 0.07–0.08, PPL 0.05–0.07, PPW 0.08–09, SI 69–88, CI 74–89 (N=7).

**Figures 4–6.**
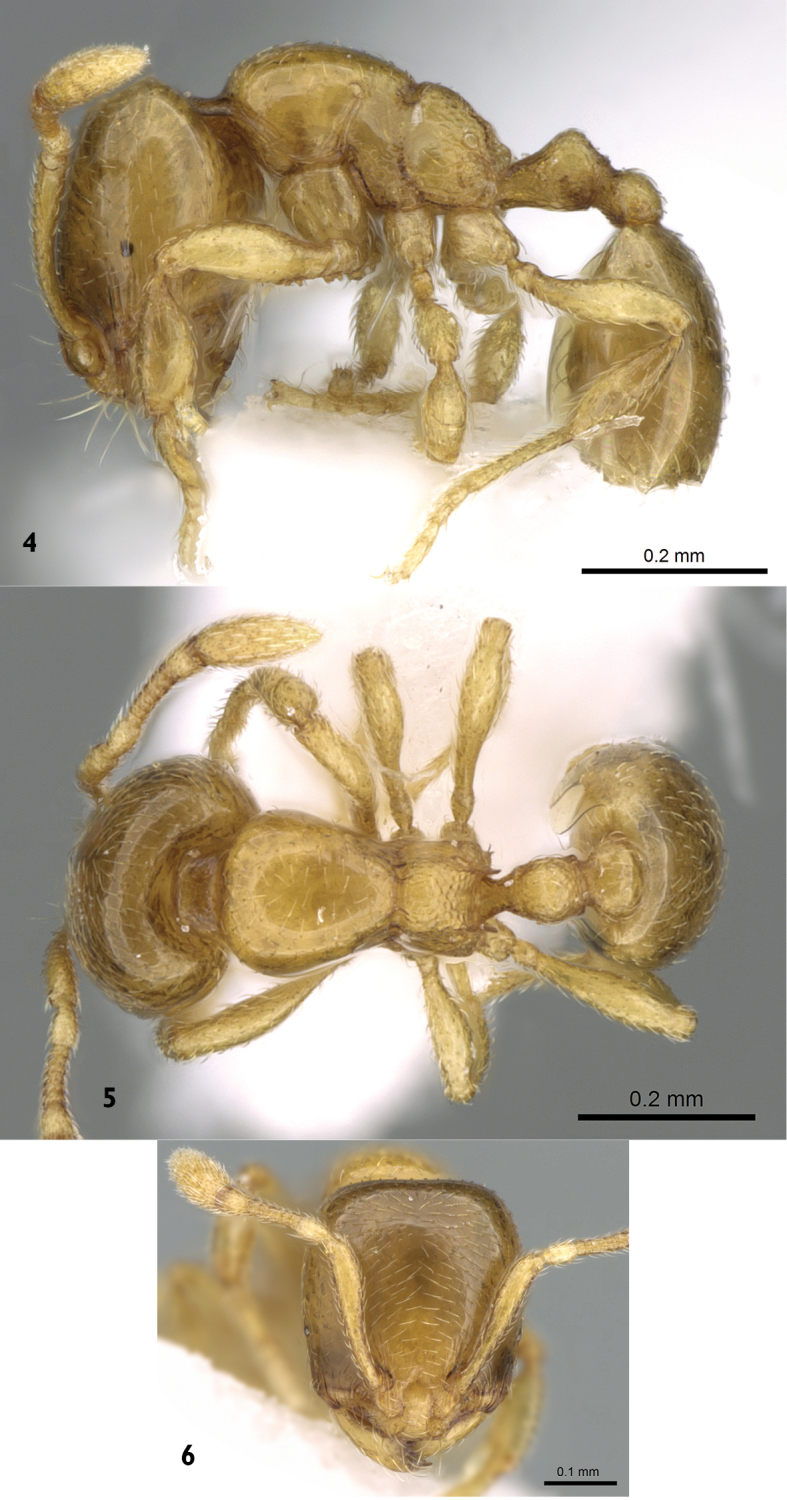
*Carebara arabica*, minor worker. **4** body in profile **5** body in dorsal view **6** head in full-face view, (antweb.org, CASENT0906368).

**Minor worker.** (Figs 4–6) Head distinctly longer than broad (CI 74-89), with clearly convex sides and straight posterior margin; mandibles smooth and shining with relatively long yellow hairs and armed with four teeth; median portion of clypeus flat; in anterolateral view, clypeal lateral carinae strongly narrowed posteriorly between frontal lobes, then continued as a frontal triangle; eyes minute, with a single ommatidium; antennae ten segmented with a two segmented club; scapes broaden evenly from about mid-length and fail to reach head posterior margin by about one-third of the head length. Mesosoma in lateral view feebly convex; metanotal groove shallow but distinct, dorsally and laterally; propodeum obliquely angled; propodeal spiracle relatively large, circular, high and close to propodeal declivity; metapleural gland orifice prominent. Petiole longer than broad in dorsal view with short peduncle. Node of postpetiolelower than petiole and dorsally clearly convex and nearly as long as broad. Sculpture: Anterolateral sides of head very finely longitudinally striated; lower half of mesopleura, metapleura, petiole and postpetiole with areolate-rugose sculpture. Pilosity: appressed, cephalic dorsum with abundant scattered hair pits, few and short on mesosoma, petiole, postpetiole, and rare on first gastral tergite, underside of head with few short straight hairs. Clypeus with two pairs of standing hairs, central pair long and lateral pair shorter. Colour: Overall unicolorous yellow, smooth and shining.

#### Remarks.

A recent search conducted by the senior author and Tony Hunter (Curator of Entomology, WMLC) failed to locate any original type material of *Carebara arabica* at the cited depository (Collingwood and van Harten 2001). Identification of this species has been difficult for non-specialists due to the brief original description and illustrations not indicating important diagnostic characters. Due to the apparent loss of all type material and the brief description, a Neotype from southwestern KSA is designated above for *Carebara arabica*.

Following the definition of Fernández (2004), *Carebara arabica* belongs to the *Carebara concinna* species complex that can be recognized by the following combination of characters: the minor workers are very small; the majors with massive heads; antennae nine to eleven-segmented, with a two segmented club; mandibles armed with four or five teeth; eyes present but reduced in both minor and major workers; metanotal groove distinct; propodeum armed with triangular teeth or denticles.

#### Biology.

*Carebara arabica* was found in Fayfa, KSA nesting in leaf litter among *Azadirachta indica* A. Juss. (Meliaceae), *Artocarpus heterophyllus* Lam (Moraceae), and *Rosadamascena* Mill. (Rosaceae) trees and coexisting with an unidentified termite species. The other nest series from Wadi Bagara was found nesting in loose soil under roots of a Poaceae and near *Acacia* and Giant Milkweed, *Calotropis procera* (Aiton) (Asclepiadaceae). Other ants’ associates included *Paratrechina jaegerskioeldi* (Mayr, 1904); *Tapinoma melanocephalum* F., 1793, and *Cardiocondyla* sp. An interesting observation concerning a nest series from Zei Ein Archaeological Village included major workers, an uncommon phenomenon as compared to other nests found in Wadi Khat, Wadi Bagara, Wadi Aljora, and Fayfa. Two major workers were collected by digging in soil. Numerous minor workers were observed foraging above ground and exiting and entering tiny nest entrances in compacted humid clay soil. No major workers were observed foraging above ground.

### Carebara fayrouzae

Sharaf sp. n.

http://zoobank.org/4E99379F-D985-4582-872A-A2B98D01F547

http://species-id.net/wiki/Carebara_fayrouzae

Figs 7 –15

#### Type material.

**Holotype major worker.** SAUDI ARABIA, Al Qatif, El Naft, Eastern Province, 26.51028°N, 49.96889°E, 30 m. 23.iii.2012 (M. R. Sharaf Leg.) (MRS0066); King Saud University Museum of Arthropods (KSMA), College of Food and Agriculture Sciences, King Saud University, Riyadh, KSA.

**Paratypes.** 28 minor workers (CASENT0280994), 16 major workers (CASENT0280975), 4 queens (CASENT0906362) with same locality and data as the holotype (KSMA); a single paratype specimen of minor and major workers, and queen are deposited in MHNG; NHMB; CASC; MCZC; SEMC; WMLC; BMNH; 2 minor workers, SAUDI ARABIA, Riyadh, Alhaeir, 24.59214°N, 46.74522°E, 24.iii.2009, lemon soil, Berlese funnel (Acarology lab team at Department of Plant Protection, KSU Leg.); 4 minor workers, SAUDI ARABIA, Riyadh, iii.1989, Soil fauna (no collector data); 2 minor workers, SAUDI ARABIA, N. Riyadh, Ammaryia, 5.iii.2010, 24.806402°N, 46.428845°E, 681m, Lettuce soil fauna, Berlese funnel (Acarology lab team at Department of Plant Protection, KSU Leg.) These paratypes are in KSMA; 1 minor worker, SAUDI ARABIA, Riyadh, Mezahmia, Rawdat Kharara, 24.38931°N, 46.24211°E, 712 m. 30.i.2011, (M. R. Sharaf Leg.) (CASC); 1 minor worker, SAUDI ARABIA, Riyadh, Al Rowayda, 25.88016°N, 45.11563°E, 22.ii.2009, (M. R. Sharaf Leg.) (CASC) (All are paratypes).

#### Description.

**Holotype major worker.** TL 3.62, HL 1.10, HW 0.72, SL 0.37, EL 0.05, ML 0.87, PRW 0.50, PL0.20, PW 0.25, PPL 0.20, PPW 0.27, SI 51, CI 65.

**Paratype major workers.** TL 3.27–5.00, HL 1.00–1.20, HW 0.72–0.87, SL 0.32–0.42, EL 0.05, ML0.85–1.12, PRW 0.50–0.60, PL 0.20–0.27, PW 0.22–0.30, PPL 0.20–0.27, PPW 0.27–0.40, SI 38–53, CI 65–78 (N=15).

**Figures 7–9.**
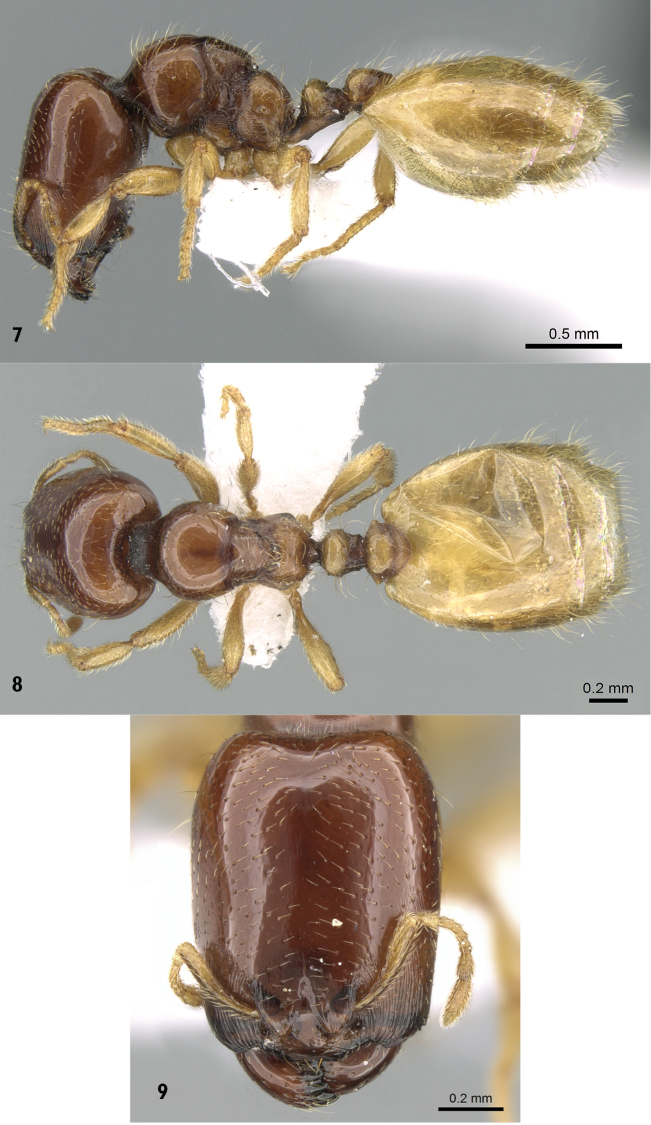
*Carebara fayrouzae* sp. n., major worker **7** body in profile **8** body in dorsal view **9** head in full-face view (antweb.org, CASENT0280975).

**Major worker.** (Figs 7–9) Head longer than broad (HL = 1.5 × HW), with feebly concave posterior margin, rounded posterior corners and parallel sides; masticatory margin of mandibles armed with four teeth; antennae nine-segmented; antennal scapes very short; clypeus narrow and with weakly concave anterior margin; eyes very tiny (in some individuals eyes absent); ocelli absent. Mesosoma in profile with distinct promesonotal suture; promesonotum feebly concave; metanotum small and narrow; dorsal face of propodeum continuously sloping and curving into declivity without spine or angle. Petiole in dorsal view little broader than long; petiolar peduncle short; petiolar ventral process distinct. Postpetiole in dorsal view nearly twice as broad as long, with acute lateral angles. Postpetiole in profile with distinct ventral process. Sculpture: Body smooth and glossy, anterior part of head finely, longitudinally rugulose, area between meso- and metapleura finely cross-ribbed. Pilosity: Head dorsum with scattered short hairs, rest of body with longer dense, yellow hairs. Colour:Bicolored species, head and mesosoma brownish, petiole and postpetiole brownish yellow, antennae, legs and gaster clear yellow.

**Paratype minor workers.** TL 1.66–1.94, HL 0.44–0.51, HW 0.29–0.44, SL 0.25–0.31, EL 0.007, ML0.49–0.53, PRW 0.22–0.31, PL 0.09–0.17, PW 0.09–0.14, PPL 0.07–0.09, PPW 0.09–0.12, SI 59–79, CI 78–100 (N=15).

**Figures 10–12.**
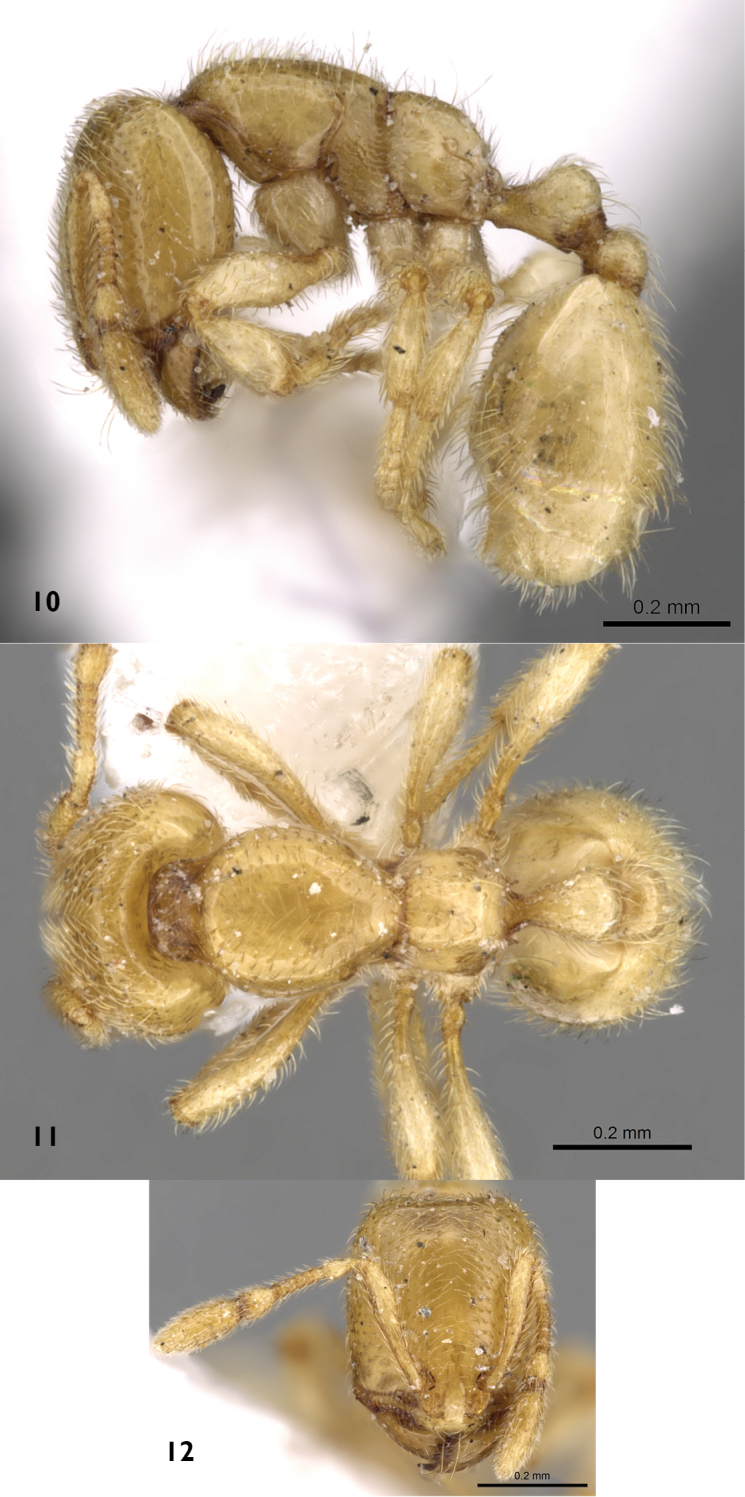
*Carebara fayrouzae* sp. n., minor worker **10** body in profile **11** body in dorsal view **12** head in full-face view (antweb.org, CASENT0280994).

**Minor worker.** (Figs 10–12) Head distinctly longer than broad, with straight posterior margin and parallel sides; masticatory margin of mandibles armed with four teeth; antennae nine-segmented; scapes when laid back from their insertions fail to reach posterior margin of head by about one third of head width; anterior clypeal margin nearly straight; eyes with only one ommatidium (absent in some individuals). Mesosoma nearly flat in profile; promesonotal suture indistinct; metanotal groove distinct; propodeal dorsum meeting declivity in a continuous curve; propodeal spiracle in profile high and located above midline of propodeum. Petiole in profile shortly pedunculate, with blunt ventral process and in dorsal view slightly longer than broad. Node of postpetiole in dorsal view clearly broader than long and in profile distinctly lower than petiolar node. Colour:Unicolorous clear yellow, hairy, smooth and moderately shining.

**Paratype queens.** TL 9.75–10.75, HL 1.10–1.35, HW 1.45–1.50, SL 0.60–0.90, EL 0.35-0.45, ML3.25–3.50, PL 0.50–0.75, PW 0.65–0.70, PPL 0.50–0.60, PPW 0.85–1.00, SI 40–62, CI 107–136 (N=3).

**Figures 13–15.**
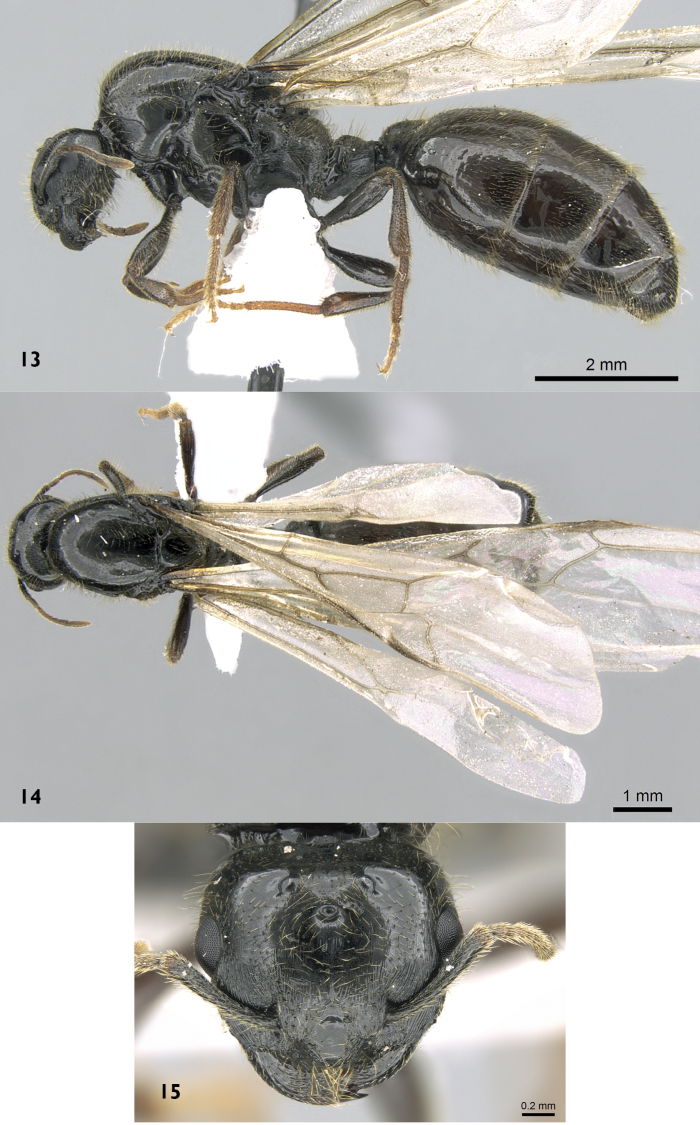
*Carebara fayrouzae* sp. n., Queen **13** body in profile **14** body in dorsal view **15** head in full-face view, (antweb.org, CASENT0906362).

**Queen.** (Figs 13–15) Body enormous, notably larger than minor and major workers. Head triangular, broader than long (HW = 1.2 × HL), with straight posterior margin and strongly convex lateral margins; masticatory margin of mandibles armed with four teeth; antennae nine-segmented; antennal scapes when laid back from their insertions reach level of posterior margin of eyes; anterior clypeal margin convex, eyes large and multifaceted (about 0.27 × HW); ocelli present. Mesosoma robust, pronotum not exposed above, lying entirely beneath the mesonotum; propodeum unarmed; remaining characters modified as in myrmicine queens. Petiole in dorsal view longer than broad. Postpetiole very broadly attached to gaster and node in dorsal view distinctly broader than long. Sculpture: Anterior half of cephalic dorsum with fine longitudinal striations extend to before posterior level of eyes; median portion of clypeus, posterior half of head, mesosoma and gaster smooth and shining, petiole and postpetiole superficially and finely shagreenate. Pilosity: Whole body covered with abundant, long, yellow hairs. Colour: Uniformly black, funiculi, tibiae and tarsi blackish brown.

**Male.** Not known.

#### Comparative notes.

This new species is the second member of the genus recorded from the Arabian Peninsula. According to Fernández (2004), *Carebara fayrouzae* belongs to *Carebara lignata* species complex with nine antennal segments and unarmed propodeum. It does not resemble any of the American species (Fernández, pers. comm.), or Madagascar species (Azorsa, pers. comm.) and majors are easily distinguished from *Carebara arabica* of the *Carebara concinna* group, by the nine segmented antennae, the absence of cephalic posterolateral teeth and the smooth and shining cephalic dorsum. Superficially, *Carebara fayrouzae* resembles *Carebara afghana* (Pisarski, 1970) from Afghanistan in regard to the smooth and shining habitus and the absence of cephalic posterolateral teeth. *Carebara fayrouzae* can be easily distinguished by the bicoloured major workers, the nine-segmented antennae and the node of postpetiole which is broader than petiolar node in dorsal view. In *Carebara afghana*, major workers are concolorous yellow, antennae ten-segmented and node of postpetiole as broad as petiolar node in dorsal view.

#### Biology.

This new species was found nesting in leaf litter under a large almond tree, *Prunus amygdalus* Batsch (Rosaceae) in a fenced area of a farm. The soil was composed of two layers, a thin upper clay layer organically enriched where most specimens were found foraging, and a lower thicker layer of loose sand where few specimens were found. Two beetle species were found coexisting with the ants, *Oryzaephilus surinamensis* (Silvanidae) and *Cryptophagus acutangulus* (Cryptophagidae).

#### Etymology.

This new species is dedicated to Fayrouz Sharaf (the daughter of the senior author).

## Discussion

The Arabian Peninsula is located on the line of contact between three major zoogeographical regions, the Palearctic, the Afrotropical and the Oriental regions; therefore, it is reasonable that it shares some faunal affinities with the mentioned regions. The central and eastern areas of the Arabian Peninsula belong to the Palearctic region with Eremic influence (Uvarov 1938, Büttiker and Wittmer 1979, Greathead 1980 and Larsen 1984, Sharaf et al. 2013); the southwestern region (Mountains of Al Sarawat and Asir to Yemen) belong to the Afrotropical region (Bodenheimer 1937, Nayman 1972, Zohary 1973, Sharaf et al. 2012 and Elhawagryi et al. 2013); whereas the north east, particularly near Iraqi and Kuwaiti borders and along mountains of eastern Oman belong to the Oriental region (Büttiker and Wittmer 1979).

In spite of the mentioned faunal affinities, the probability of *Carebara fayrouzae* to be an introduced or invasive species, is unlikely for two reasons, first, the new species is well represented very much inland in central Arabian deserts (Riyadh and adjacent areas), second, it does not resemble any of the Asian species, (*e.g.* the Indian species) (Bharti and Kumar 2013). The poverty of knowledge of the genus for adjacent countries east of the Arabian Gulf (*e.g.* Iran, Pakistan, etc.) makes the hypothesis difficult to test completely.

At present, two species of *Carebara* are now known from the Arabian Peninsula, *Carebara arabica* of the *concinna* species complex from the Republic of Yemen and KSA and *Carebara fayrouzae* sp. n. of the *lignata* species complex from KSA. The subterranean nature and nesting habit in decaying wood or leaf litter of this ant group (Bolton 1973, Hölldobler and Wilson 1990, Bharti and Kumar 2013) no doubt has resulted in a paucity of information available about its ecology and biology (Aldawood et al. 2011, Bharti and Kumar 2013). It seems likely that *Carebara arabica* is a species of mountainous ecosystems of southwestern KSA and Republic of Yemen. In contrast, *Carebara fayrouzae* was found in desert ecosystems of the central and eastern regions of KSA.

## Supplementary Material

XML Treatment for Carebara arabica

XML Treatment for Carebara fayrouzae

## References

[B1] Aldawood AS Sharaf MR Taylor B (2011) First record of the Myrmicine ant genus *Carebara* Westwood, 1840 (Hymenoptera, Formicidae) from Saudi Arabia with description of a new species, *C. abuhurayri* sp. n. ZooKeys 92: 61-69. doi: 10.3897/zookeys.92.770 PMC3084545 21594112

[B2] Arnold G (1916) A monograph of the Formicidae of South Africa. Part II. Ponerinae, Dorylinae. Annals of the South African Museum 14: 159-270.

[B3] Bharti H Kumar R (2013) Six New Species of *Carebara* Westwood (Hymenoptera: Formicidae) with Restructuring of World Species Key to Indian Species. Journal of Entomological Research Society 15: 47-67.

[B4] Bingham CT (1903) The fauna of British India, including Ceylon and Burma. Hymenoptera, Vol. II. Ants and Cuckoo-wasps. Taylor and Francis, London, 506 pp.

[B5] Bodenheimer FS (1937) Problems of animal distribution in Arabia. Proceedings of the Linnean Society of London 148: 47-48.

[B6] Bolton B (1973) The ant genera of West Africa: a synonymic synopsis with keys (Hymenoptera: Formicidae). Bulletin of the British Museum (Natural History), Entomology 54: 263–452.

[B7] Bolton B (2012) An Online Catalogue of the Ants of the World. http://www.antcat.org/ [accessed 6.VII.2013]

[B8] Bolton B Alpert G Ward PS Naskrecki P (2006) Bolton’s Catalogue of the Ants of the World: 1758–2005. Compact Disc Edition, Harvard University Press.

[B9] Brown WL Jr (2000) Diversity of ants. In: Agosti et al. (Eds) Ants. Standard methods for measuring and monitoring biodiversity. Biological diversity hand book series. Smithsonian Institution Press, Washington and London, 280 pp.

[B10] Büttiker W Wittmer W (1979) Entomological Expedition of the Natural History Museum, Basle to Saudi Arabia. Fauna of Saudi Arabia I: 23–29.

[B11] Collingwood CA van Harten A (2001) Additions to the ant fauna of Yemen. Esperiana. Buchreihe zur Entomologie 8: 559-568.

[B12] Eig A (1938) Taxonomic Studies on the Oriental Species of the Genus *Anthemis*. Palestine Journal of Botany, Jerusalem 1: 161-224.

[B13] Elhawagryi MS Khalil MW Sharaf MR Fadl HH Aldawood AS (2013) A preliminary study on the insect fauna of Al-Baha Province, Saudi Arabia, withdescriptions of two new species. Zookeys 274: 1-88. doi: 10.3897/zookeys.274.4529 PMC3677392 23794807

[B14] Ettershank G (1966) A generic revision of the world Myrmicinae related to *Solenopsis* and *Pheidologeton* (Hymenoptera: Formicidae). Australian Journal of Zoology 14: 73-171. doi: 10.1071/ZO9660073

[B15] Fernández F (2004) The American species of the myrmicine ant genus *Carebara* Westwood. Caldasia 26: 191-238.

[B16] Greathead DJ (1980) Insects of Saudi Arabia. Diptera, Fam. Bombyliidae. Fauna of Saudi Arabia 2: 291-337.

[B17] Guénard B Dunn RR (2012) A checklist of the ants of China. Zootaxa 3358: 1-77.

[B18] Hölldobler B Wilson EO (1990) The ants. Harvard University Press, Massachusetts, 732 pp. doi: 10.1007/978-3-662-10306-7

[B19] Larsen TB (1984) Butterflies of Saudi Arabia and its neighbours. Stacey International, London, 160 pp.

[B20] Longino JT (2004) Ants of Costa Rica. http://www.evergreen.edu/ants/genera/Carebara [accessed 13.X.2010]

[B21] Nayman J (1972) Atlas of Wildlife. Heinenmann, London, 124 pp.

[B22] Sharaf MR Aldawood AS Taylor B (2012) A new ant species of the genus *Tetramorium* Mayr, 1855 (Hymenoptera: Formicidae) from Saudi Arabia, Including a revised key to the Arabian species. PLoS ONE 7(2): e30811. doi: 10.1371/journal.pone.0030811 22389667 PMC3289629

[B23] Sharaf MR Abdeldayem MS Aldhafer H Aldawood AS (2013) The ants (Hymenoptera: Formicidae) of Rawdhat Khorim nature preserve, Saudi Arabia, with description of a new species of the genus *Tetramorium* Mayr. Zootaxa 3709(6): 565-580. doi: 10.11646/zootaxa.3709.6.6 26240931

[B24] Shattuck SO (1999) Australian ants. Their biology and identification. CSIRO Publishing, Collingwood, Victoria, xi + 226 pp.

[B25] Taylor B (2010) http://www.webarchive.org.uk/wayback/archive/20100617220031/, http://antbase.org/ants/africa/ [accessed 04-28-2013]

[B26] Taylor RW Brown DR (1985) Formicoidea. Zoological Catalogue of Australia 2: 1–149, 306–348.

[B27] Terayama M (1996) Taxonomic studies on the Japanese Formicidae, part 2. Seven genera of Ponerinae, Cerapachyinae and Myrmicinae. Nature & Human Activities 1: 9-32.

[B28] Terayama M (2009) A synopsis of the family Formicidae of Taiwan (Insecta: Hymenoptera). Research Bulletin of Kanto Gakuen University. Liberal Arts 17: 81-266.

[B29] Uvarov BP (1938) Ecological and biogeographical relations of Eremian Acrididae. Mémoires de la Société de Biogéographie de Paris 6: 231-273.

[B30] Weber NA (1950) The African species of the genus *Oligomyrmex* Mayr. American Museum Novitates 1442: 1-19.

[B31] Xu Z (1999) Systematic studies on the ant genera of *Carebara*, *Rhopalomastix* and *Kartidris* in China. Acta Biologica Plateau Sinica 14: 129-136.

[B32] Zohary M (1973) Geobotanical foundations of the Middle East, vols. 1–2. Fischer, Stuttgart, Amsterdam, Swets, Zeitlinger, 738 pp.

